# Adult breast, lung, pancreatic, upper and lower gastrointestinal cancer patients with hospitalized venous thromboembolism in the national French hospital discharge database

**DOI:** 10.1186/s12885-023-10877-4

**Published:** 2023-06-10

**Authors:** F. Couturaud, I. Mahé, J. Schmidt, J-C. Gleize, T. Lafon, A. Saighi, F. Sedjelmaci, L. Bertoletti, P. Mismetti

**Affiliations:** 1 Univ Brest, INSERM U1304-GETBO, Département de médecine interne et pneumologie, CHU Brest, Brest, France; 2 FCRIN INNOVTE, Saint-Etienne, France; 3 Université de Paris, APHP, Hôpital Louis Mourier, Service de Médecine Interne, Colombes, France; 4 Innovative Therapies in Haemostasis, INSERM, Paris, France; 5 CHU de Clermont-Ferrand, Clermont-Ferrand, France; 6 HEVA, Lyon, France; 7 PFIZER, Paris, France; 8 Service de Médecine Vasculaire Et Thérapeutique, CHU de St-Etienne, Saint-Etienne, France; 9 INSERM, UMR1059, Université Jean-Monnet, Saint-Etienne, France; 10 INSERM, CIC-1408, CHU de Saint-Etienne, Saint-Etienne, France

**Keywords:** Venous thromboembolism, Cancer, Epidemiology, Mortality

## Abstract

**Background:**

Venous thromboembolism (VTE) and cancer are strongly associated. In France, evidence on patients with pancreatic, upper GI [gastrointestinal], lower GI, lung, or breast cancer-associated VTE and their hospital management is limited. The aims of this study were to provide data on the number of hospitalized VTE events among cancer patients, the patients’ characteristics, and their hospital management to estimate the burden of disease and the hospital burden of cancer-related VTE and to provide guidance on research.

**Methods:**

This longitudinal, observational, and retrospective study was based on the comprehensive hospital discharge database (PMSI). Adult patients (≥ 18 years old) hospitalized with a cancer of interest in 2016 and hospitalized (within 2 years with VTE (captured a as a principal, related, or significant associated diagnosis) were included in the study.

**Results:**

We identified 340,946 cancer patients, of which 7.2% (24,433 patients) were hospitalized with VTE. The proportions of hospitalized VTE were 14.6% (3,237) for patients with pancreatic cancer, 11.2% (8,339) for lung cancer, 9.9% (2,232) for upper GI cancer, 6.7% (7,011) for lower GI cancer, and 3.1% (3,614) for breast cancer. Around two thirds of cancer patients with a hospitalized VTE had active cancer (with metastases and/or receiving chemotherapy during the six months prior to the index date): from 62% of patients with pancreatic cancer to 72% with breast cancer.

Around a third of patients were admitted to the hospital through the emergency room, up to 3% of patients stayed in an intensive care unit. The average length of stay ranged from 10 (breast cancer) to 15 days (upper GI cancer). Nine (lower GI cancer) to 18% (pancreatic cancer) of patients died during the VTE hospital stay.

**Conclusions:**

The burden of cancer-associated VTE is substantial, both in terms of the number of patients affected and in the hospital use. These findings offer guidance on future research on VTE prophylaxis in a very high-risk population, particularly in patients with active cancer.

**Supplementary Information:**

The online version contains supplementary material available at 10.1186/s12885-023-10877-4.

## Introduction

Venous thromboembolism (VTE) and cancer are both major burdens of disease in France. In 2010, more than 120,000 individuals were hospitalized with a VTE event and more than 14,800 patients died of VTE [1]. In 2018, 382,000 cancer cases were diagnosed [2].

Cancer and VTE are strongly associated, yet there is a lack of data on cancer patients experiencing VTE in France. Reasons for this close connection include VTE risk factors in cancer patients: older age, comorbidities which may affect the efficacy and safety of anticoagulant treatments, immobility, and prior history of VTE [3–5]. Then, some cancer-related factors increase the risk of VTE: some cancer sites, advanced stage [3–6], and treatments (surgery, chemotherapy, and the use of central venous catheters) [7, 8]. For instance, data displayed the highest rates of VTE in pancreatic, lung, and gastrointestinal (GI) cancer patients [9–11]. In women, higher rates of VTE are also found in ovarian and breast cancer patients [9, 10]. Furthermore, active cancer is also a predictor of VTE recurrence [12–14].

Patients with cancer-associated thrombosis (CAT) represent a significant challenge since they are at higher risk of both VTE recurrence and major bleeding compared to patients without cancer [15, 16]. Treatment guidelines have considerably changed over the recent years and currently rely upon low-molecular weight heparin (LMWH) and direct oral anticoagulants such as apixaban, rivaroxaban or edoxaban [17–19].

The aims of our study were to fill the evidence gap regarding CAT in France and to provide up-to-date data on the number of severe VTE events among cancer patients, the patient characteristics, and their hospital management. As the VTE events of interest were the severe VTE events and current guidelines require these events to be managed at the hospital [20, 21], the French hospital discharge database (a nationwide, comprehensive claims database) was evaluated. We present the top 3 tumors with the highest VTE rate (pancreas, lung, and upper GI), as well as lower GI and breast cancer for the large number of VTE among these patients. Our study presents a snapshot of the cancer patients who were hospitalized for their cancer in 2016 followed by a hospitalized VTE event within two years of this 2016 cancer-related hospital stay. VTE events examined in our study encompass deep venous thrombosis and all-location embolism (including obstetric embolism).

## Methods

### Study design and data sources

This longitudinal, observational, and retrospective study was based on the hospital discharge database (PMSI, Programme de Médicalisation des Systèmes d’Information) of the National Health Data System (SNDS, Système National des Données de Santé) [22]. It contains comprehensive data on healthcare resource consumption of all hospital stays in France (both in the public and private sectors). Specifically, this study evaluated the PMSI-MCO (Medicine-Surgery-Obstetric) dataset, which provides reason for hospitalization, medical procedures, comorbidities which required care during the hospital stay, and medical devices implant.

The PMSI-MCO contains the hospital discharge forms of all hospital stays. In those forms, the reasons and related motives for the hospital stay are codes with the ICD-10. The principal diagnosis (PD) is the main reason for the hospitalization. The related diagnosis/diagnoses (RD) is/are reason/s contributing to the PD or qualifying the PD (when the PD is a specific procedure or health care). The significant associated diagnosis/diagnoses (SAD) indicate comorbidities that are relevant for the patient’s therapeutic management or diseases occurring during the hospital stay.

Of note, the PMSI does not provide information on drugs that are part of the standard care, such as anticoagulants, as they are included in the lump sum paid by the national health insurance to the hospital for the hospital stay. However, information about innovative and expensive drugs (called “liste en sus”) (e.g. immunotherapies) administered to patients are available.

Cancer patients are treated at the local hospital. Patients living in large cities can be treated in regional reference centers.

The reliability of the PMSI-MCO depends on the healthcare providers' ability to accurately code all relevant diagnoses during a hospital stay. In the study of Prat and colleagues, the positive predictive value (PPV) of codes in the PMSI was 95.0% (95% CI 88.2–98.1%) for peripheral venous thrombosis, 99% (CI 95% 93.8–99.9%) for pulmonary embolism, and 85% (95% CI 76.7–90.7%) for thrombosis location [23].

### Study population and study period

The patient selection was a two-step process. First, all adult patients (≥ 18 years old) hospitalized with a cancer of interest (identified with the PD, the RD or the SAD in the hospital discharge form) in France between January 1, 2016, and December 31, 2016, were identified and used as a reference population. Second, among them, only cancer patients with a hospital stay with a VTE ICD-10 code within two years of the cancer-related hospital stay were included in the study (identified with the PD, the RD, or the SAD in the hospital discharge form). The inclusion hospital stay was the 2016 cancer-related hospital stay; the index hospital stay was the first VTE-related hospital stay following the inclusion hospital stay. All hospitalized VTE event within two years after the inclusion hospital stay were analyzed. The two-year follow-up allowed to detect hospitalized VTE in cancers with lower VTE rates (breast) and in smaller cancer populations (upper GI and pancreatic cancer).

In addition, a follow-back period of five years before the inclusion hospital stay was used to capture any prior cancer-related hospital stay which could inform on the time since the cancer was diagnosed. This period was limited to three months prior to the VTE event when searching for relevant recent clinical events (i.e., hospitalization for an acute medical or surgical event).

The cancers of interest were those located in the upper and lower GI tract, pancreas, lung, and breast. Patients were included if they presented with carcinoma in situ or invasive cancer (ICD-10 codes in Supplement Table 1).

### Outcomes

The primary outcome was hospitalized VTE identified based on the following ICD-10 codes: I26*, I63.6, I67.6, I80*, I82*, O22.2–3 and O22.5, O87.0–1 and O87.3, and O88.2 during the two-year follow-up after cancer identification (Supplement Table 2). All embolism codes in the hospital discharge form were considered: I26.0, I26.9 (pulmonary embolism), I82.2–3, I82.8–9 (embolism of veins), and O.88.2 (obstetric embolism) (Supplement Table 2).

### Data collection

In this descriptive study, the variables of interests were the patient characteristics (age, sex, recent clinical events in the past three months), characteristics of their cancer (active or not, presence of metastases or not), and the number of subsequent hospitalized VTE events. The proportion of patients with VTE among cancer patients was calculated for each cancer of interest.

The patients’ characteristics were described for cancer patients at the time of inclusion, and for cancer patients experiencing a VTE event managed at the hospital, at the time of their first VTE-related hospital stay. Collected clinical events in the PMSI prior to VTE were surgery, trauma, stroke, heart failure, respiratory failure, and infection, all of which were identified through ICD-10 codes.

Patients with active cancer were defined as patients with metastases and/or receiving chemotherapy during the six months prior to the index date. The presence of metastases was sought without limitation on the whole extraction period (i.e., 5 five years prior and two years after the inclusion hospital stay) through the presence of metastases ICD-10 codes (C77, C78 and C79). We acknowledged that the development of metastases reported after the index date may have already started earlier and might have contributed to the VTE event.

The time since diagnosis of cancer was defined by the proxy variable of the interval between the first hospitalization for cancer and the index VTE date, as the exact date of diagnosis is not documented in the PMSI.

The index and subsequent hospital stays were also described in terms of visited departments, admission and discharge modes, hospital stay duration and medical procedures performed during the hospital stay. The visited departments were markers of severity for the patient with in increasing severity order: continuous monitoring unit, intensive care unit and resuscitation unit. The arrival mode was either through the emergency department or not, and the discharge mode was transfer to another unit or hospital, discharge to home or death. Medical procedures of interest performed during the stay were identified through CCAM procedure codes (Classification Commune des Actes Médicaux) (Supplement Table 3) and included vena cava filter insertion, fibrinolysis, thromboaspiration, and thrombectomy.

### Statistical methods

Continuous data were expressed as mean, standard deviation (SD), median, first and third quartiles. Categorical data were summarized by frequency and percentage of patients in each category. The proportion of VTE events among cancer patients were compared across cancers with two-sided Chi^2^ tests at a 5% significance level.

The statistical analyses were performed with SAS software (version 9.4).

## Results

### Study population

**Fig. 1:**
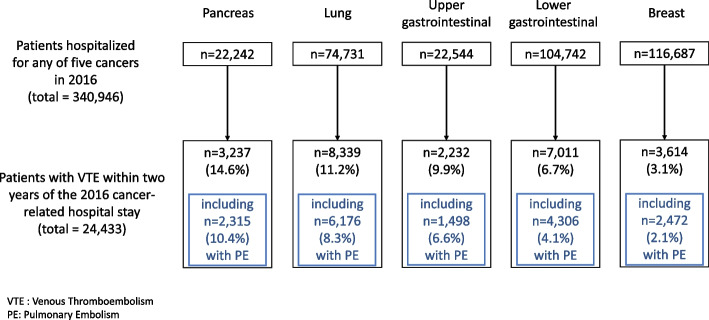
Study population. The proportion of patients with a VTE was statistically different between cancers (*p* < .0001 for each chi^2^ test comparing cancers two-by-two)

Between January 1^st^, 2016, and December 31^st^, 2016, 340,946 patients were hospitalized with one of the five cancers of interest: 116,687 patients were hospitalized with breast cancer, 104,742 patients were hospitalized with lower GI cancer, 74,731 patients were hospitalized with lung cancer, 22,544 patients were hospitalized with upper GI cancer, and 22,242 patients were hospitalized with pancreatic cancer (Fig. 1).

Those patients were followed-up during two years after their 2016 inclusion hospital stay. Of these 340,946 cancer patients, 24,433 (7.2%) experienced a VTE-related hospital stay within the two years of their 2016 cancer-related hospital stay. The proportion of patients experiencing a hospitalized VTE was highest among pancreatic cancer patients (14.6%, 3,237 patients), followed by lung cancer patients (11.2%, 8,339 patients), upper GI cancer patients (9.9%, 2,232 patients), then lower GI cancer patients (6.7%, 7,011 patients), and finally breast cancer patients (3.1%, 3,614 patients). The proportion of patients with a VTE was statistically different between cancers (*p* < 0.0001 for each chi^2^ test comparing cancers two-by-two). Not only the proportion of patients with a hospitalized VTE was highest among pancreatic cancer patients, but they also had the highest proportion of hospitalized all-location embolism (10.4%) —which was counted as a hospitalized VTE event.

### Patients’ characteristics

Among all cancer patients, the mean (SD) age at inclusion varied from 61.1 years (± 13.6) among breast cancer patients to 69.7 years (± 12.0) among pancreatic cancer patients (Table 1). The mean age at index date (first VTE event) among cancer patients with a hospitalized VTE was higher than the mean age of all cancer patients at inclusion (i.e., whether they had VTE or not) among breast and lower GI cancers patients (65.6 vs 61.1 years old and 70.4 vs 68.5 years old, respectively),It wasone year lower among pancreatic and lung cancer patients (68.7 vs 69.7 years old and 65.5 vs 66.5 years old, respectively), and almost identical among upper GI cancer patients (around 68.3 years). For each cancer, the proportion of women among cancer patients with a hospitalized VTE was similar to the proportion of women among the whole population of cancer patients.

**Table 1:** Patient characteristics

Cancer	Pancreas	Lung	Upper gastrointestinal	Lower gastrointestinal	Breast
Age, mean (± SD) median [1^st^ and 3^rd^ quartiles], years					
All cancer patients, at inclusion hospital stay (*n* = 340,946)	69.7 (± 12.0)	66.5 (± 10.9)	68.3 (± 12.5)	68.5 (± 12.3)	61.1 (± 13.6)
	70 [62–78]	66 [59–74]	68 [60–78]	69 [61–77]	61 [51–71]
Cancer patients with a hospitalized VTE, at index hospital stay (*n* = 24,433)	68.7 (± 11.2)	65.5 (± 10.9)	68.2 (± 12.3)	70.4 (± 12.1)	65.6 (± 13.8)
	69 [62–77]	65 [58–73]	69 [60–77]	71 [63–80]	66 [56–76]
Sex, female (%)					
All cancer patients (*n* = 340,946)	10,841 (49%)	23,951 (32%)	6,630 (29%)	46,431 (44%)	115,540 (99%)
Cancer patients with a hospitalized VTE (*n* = 24,433)	1,592 (49%)	2,848 (34%)	650 (29%)	3,114 (44%)	3,545 (98%)
Time between first cancer-related hospital stay^a^ and index VTE hospital stay, mean (± SD) median [1^st^ and 3^rd^ quartiles], months					
Cancer patients with a hospitalized VTE (*n* = 24,433)	8.7 (± 12.9)	10.9 (± 15.3)	10.6 (± 14.3)	14.7 (± 18.8)	21.1 (± 21.5)
	3.4 [0–12.9]	4.6 [0.1–16.2]	5.3 [1.1–15.3]	7.2 [0.9–21.2]	14.7 [3.3–33.9]
Cancer status, active^b^ (%)					
All cancer patients, at inclusion hospital stay (*n* = 340,946)	14,926 (67%)	46,803 (63%)	14,305 (63%)	48,793 (47%)	61,948 (53%)
Cancer patients with a hospitalized VTE, at index hospital stay (*n* = 24,433)	2,023 (62%)	5,930 (71%)	1,451 (65%)	4,446 (63%)	2,584 (72%)
Presence of metastases, yes (%)					
All cancer patients, at inclusion hospital stay (*n* = 340,946)	7,838 (35%)	31,198 (42%)	8,578 (38%)	36,891 (35%)	41,042 (35%)
Cancer patients with a hospitalized VTE, at index hospital stay (*n* = 24,433)	1,279 (40%)	4,374 (52%)	1,081 (48%)	3,537 (50%)	2,036 (56%)
Clinical events during the three months prior to the hospitalized VTE (*n* = 24,433)					
Infections^c^	432 (13%)	1214 (15%)	333 (15%)	693 (10%)	319 (9%)
Surgery	280 (9%)	899 (11%)	308 (14%)	1214 (17%)	555 (15%)
Trauma	54 (2%)	199 (2%)	36 (2%)	157 (2%)	77 (2%)
Respiratory failure	48 (1%)	361 (4%)	72 (3%)	81 (1%)	52 (1%)
Heart failure	14 (< 1%)	57 (1%)	6 (< 1%)	30 (< 1%)	16 (< 1%)
Stroke	12 (< 1%)	66 (1%)	6 (< 1%)	13 (< 1%)	8 (< 1%)

*VTE* venous thromboembolism, *SD* standard deviation

^a^First cancer-related hospital stay: first cancer-related hospital stay within five years prior to the 2016 inclusion cancer-related hospital stay

^b^Active cancer: presence of an ICD-10 code indicative of metastases during the two-year follow-up period, or a chemotherapy within the six months prior to the VTE event

^c^Infections requiring hospitalization and occurring during a hospital stay

The mean time (SD) between the earliest recorded cancer-related hospital stay (within the five years prior to the index hospital stay) and the index hospital stay in cancer patients with a VTE ranged from 8.7 months (± 12.9) for pancreatic cancer patients to 21.1 months (± 21.5) for breast cancer patients. Half of the hospitalized VTE events occurred within the first six months after the earliest recorded cancer-related hospital stay in pancreatic, lung and upper GI cancer patients (3.4, 4.6 and 5.3 months, respectively).

**Fig. 2:**
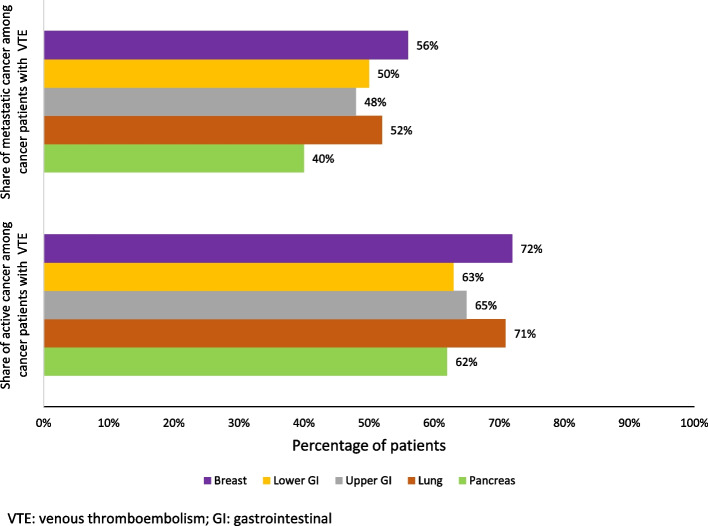
Proportion of patients with venous thromboembolism in cancer patients, with active cancer in cancer patients with VTE and with a metastatic cancer in cancer patients with VTE, by cancer

Around two thirds of cancer patients with a hospitalized VTE had active cancer (from 62% for pancreas cancer to 72% for breast cancer, Fig. 2, Table 1). The proportion of active cancer was 2 to 19 percentage points higher in patients experiencing a hospitalized VTE than in the corresponding whole population of cancer patients (but 5% lower for pancreatic cancer, Table 1). About half of the patients with cancer and a hospitalized VTE had metastases: from 40% for pancreas cancer to 56% for breast cancer. The proportion of patients with metastatic cancer and a hospitalized VTE was 5 to 20 percentage points higher than the proportion of patients with metastatic cancer in the whole population (40–56% vs 35–42%).

Two clinical events were more frequently detected during the three months prior to the event: infections requiring a hospital stay or occurring during a hospital stay (in 9–15% of patients) and surgery (in 9–17% of patients) (Table 1).

### Hospital management of the VTE-related hospital stays

Regardless of the cancer, around a third of patients arrived at the hospital for their VTE-related hospital stay through the emergency room (Table 2). The duration of a VTE-related hospital stay (initial and subsequent hospital stays combined) was between 10 days for breast cancer to 15 days for upper GI cancer. Regardless of the cancer, less than 3% of patients were admitted to the resuscitation department, the intensive care unit, or were under continuous monitoring.

**Table 2:** Hospital management of cancer patients with venous thromboembolism, during (initial and subsequent) venous thromboembolism-related hospital stays

Cancer	Pancreas	Lung	Upper gastrointestinal	Lower gastrointestinal	Breast
Arrival at the hospital through the emergency room, yes (%)	1,495 (32%)	4,207 (35%)	974 (32%)	3,166 (34%)	1,666 (35%)
Duration of the index hospital stay					
mean (± SD), days	12.0 (± 13.3)	11.8 (± 13.8)	15.2 (± 18.9)	13.1 (± 16.1)	10.1 (± 12.9)
median [1^st^ and 3^rd^ quartiles], days	8 [3-18]	8 [3-16]	10 [3-20]	8 [3-17]	6 [2-13]
Hospital departments visited, n (%)					
Resuscitation department	9 (< 1%)	48 (1%)	13 (1%)	49 (1%)	11 (< 1%)
Intensive care unit	55 (2%)	221 (3%)	58 (3%)	172 (3%)	101 (3%)
Continuous monitoring	49 (2%)	157 (2%)	52 (2%)	177 (3%)	47 (1%)
Procedures, n (%)					
Vena cava filter insertion	120 (4%)	248 (3%)	120 (5%)	261 (4%)	56 (2%)
Fibrinolysis	6 (< 1%)	38 (< 1%)	12 (1%)	39 (1%)	8 (< 1%)
Thromboaspiration	5 (< 1%)	19 (< 1%)	10 (< 1%)	2 (< 1%)	3 (< 1%)
Thrombectomy	4 (< 1%)	5 (< 1%)	5 (< 1%)	2 (< 1%)	0 (0%)
None of the above	3,102 (96%)	8,029 (96%)	2,085 (93%)	6,707 (96%)	3,547 (98%)
Discharge mode, n (%)					
Sent home	2,902 (63%)	7,813 (64%)	1,925 (62%)	6,584 (70%)	3,375 (70%)
Transferred to another unit or hospital	883 (19%)	2,545 (21%)	639 (21%)	1,996 (21%)	993 (21%)
In hospital mortality	839 (18%)	1,842 (15%)	521 (17%)	855 (9%)	465 (10%)

*SD* standard deviation

Resuscitation department: intended for patients who present or are likely to present several acute visceral failures directly involving the vital prognosis and requiring the use of substitution methods

Intensive care unit: intended for patients who present or are likely to present one acute failure of the organ concerned by the specialty under which they are treated, directly affecting their vital prognosis in the short term, and involving the use of a method of substitution

Continuous monitoring: intended for patients who, because of the seriousness of their condition or the treatment applied to them, require repeated and systematic clinical and biological observation

From two percent (breast cancer) to seven percent (upper GI cancer) of the cancer patients underwent any of the following four procedures: vena cava filter insertion, fibrinolysis, thromboaspiration, or thrombectomy. The most frequent procedure was the vena cava filter insertion, occurring in 2% of breast cancer patients and up to 5% of upper GI cancer patients.

Around two thirds of the patients returned home at the end of their VTE-related hospital stay: from 62% for upper GI cancer to 70% for lower GI cancer and breast cancer. The percentage of patients who died during their VTE-related hospital stay varied from 9% for those with lower GI cancer to 18% for those with pancreatic cancer. As for the other patients, they were sent home or transferred to another unit or another hospital.

## Discussion

This study involved 340,946 cancer patients hospitalized for their cancer in 2016, among which 24,433 (7.2%) were hospitalized with a VTE event within two years. The hospitalized VTE rate ranged from 3% in breast cancer patients to 15% in pancreatic cancer patients. Hospital data confirmed the urgency and severity of VTE in cancer patients, with around a third of patients admitted to the hospital through the emergency room, up to 3% of patients staying in the resuscitation department, or the intensive care unit, or under continuous monitoring, with a mean hospital stay duration of 10 (breast cancer) to 15 days (upper GI cancer), and 9% (lower GI cancer) to 18% (pancreatic cancer) of patients dying during the hospital stay.

In the absence of a nationwide study on hospitalized VTE in French cancer patients, we can measure up our results of 24,433 cancer patients hospitalized with a VTE within two years from 2016 to the more than 120,000 patients hospitalized with a VTE in 2010 in the French population [1]. Although the VTE identification criteria were slightly different from our study (ICD-10 codes I26, I80, I81 and I82), this confirms the large proportion of cancer patients among individuals experiencing a hospitalized VTE due to the higher risk of VTE in this patient population. Besides, a recent Danish study showed that the risk of VTE in cancer patients has been increasing steadily over the past two decades, parallel to the ascent of new treatments (target therapies), improved survival, and expanded use of computed tomography scans [24]. This means that the overall number of VTE events has also probably increased over time. In Denmark, cumulative incidence of VTE 12 months after the cancer diagnosis/index date was 2.3% (95% CI 2.2% – 2.3%) in the cancer cohort and 0.35% (95% CI 0.34% – 0.36%) in the comparison cohort (Hazard Ratio 8.5; 95% CI, 8.2–8.8).

Our study confirms earlier findings that pancreatic and lung cancer patients are at the highest risk of hospitalized VTE, 98 to 110 per 1,000 person-years and 44 to 45 per 1,000 person-years respectively [10, 11, 25–27]. Furthermore, surgery and infection are relevant CAT risk factors, detected in around 25% of the cases within the three months prior to a hospitalized VTE, consistent with the information found in the cancer population [8]. Those risks factors are found in an already at-risk population, as cancer is known to be an independent and major risk factor for VTE [28]. Lastly, the high proportion of hospitalized all-location embolism among hospitalized VTE events (69% overall) could be due to the hospital data collection as it was also the case in a previous PMSI analysis (62% of pulmonary embolism) [1].

Our study has several strengths worth mentioning. First, we aimed to be thorough in our patient selection. The cancer patients selection relied on an algorithm provided by the French National Healthcare Insurance (CNAM) [29] using cancer-related ICD-10 codes as a PR or RD in the hospital discharge and extended the search of the code to the SAD. Additionally, in identifying VTE events, we used a broader approach than earlier studies by including all-location embolisms. Second, the discharge database we relied on also ensures the accurate identification of the study population and prevents patient selection bias via a retrospective data collection. Finally, the PMSI-MCO captures every hospital stay and has a national coverage. Real-world studies are necessary, as clinical trials do not represent the full spectrum of cancer-associated VTE patients.

In spite of the many advantages of the PMSI-MCO, only the hospital management is covered. This means that VTE managed outside of the hospital could not be considered. However, this should not be a limitation in our study as it is required to manage all severe VTE events at the hospital [20, 21]. Moreover, due to its status of claims database, drug management of hospitalized VTE patients was unavailable, nor were the detailed clinical outcomes and the results of medical tests undergone by patients. In particular, anticoagulant dispensing —which is the main treatment for VTE among cancer patients [28, 30]— was not available. Furthermore, it is not possible to establish an association between the VTE and the cancer diagnosis or treatment in the PMSI-MCO. Regarding hospitalized VTE patients’ selection, we also could not benefit from a previously published identification algorithm and hence we developed one. With our algorithm, we captured patients with a VTE reported as SAD in the hospital discharge database. Thus, admission through the emergency department, treatment in an intensive care unit, median hospital stay duration, and in-hospital mortality may not be only caused by the VTE diagnosis. Because it is not possible to distinguish non-treated patients from patients with non-active cancer in the PMSI, active cancer status was defined as the presence of metastases or administration of chemotherapy six months prior to the index date. Disregarding other treatments (e.g., radiotherapy) has likely underestimated the number of patients with active cancer. Given that the PMSI does not record outpatient deaths and the exact date of the VTE diagnosis was unknown (with the first day of the hospital stay used as proxy), it was not possible to perform survival analyses to accurately compute a VTE mortality rate. This study fails to consider VTE in the list of recent clinical events although cancer patients with a history of VTE have a six to seven-times higher risk of VTE recurrence compared to cancer patients without VTE history [7, 31]. Finally, when analyzing the median time between the first cancer-related hospital stay and the index VTE hospital stay, a point was raised whether a two-year follow-up was sufficient to capture late VTE occurrences. Several studies in Western countries have reported that the risk of VTE event was highest three [27] to twelve months after cancer diagnosis [6] and during the first two months after chemotherapy or surgery among breast cancer patients [32]. Hence, a follow-up period of two years was deemed sufficient.

## Conclusion

This nationwide study identified around 340,000 French patients with five common cancers (pancreas, lung, upper/lower GI, and breast). Over the two-year follow-up period, about 25,000 of these cancer patients experienced at least one hospitalized VTE, i.e., between 3 and 15% of all cancer patients, depending on the cancer. Our results allowed to update and confirm the substantial burden of CAT in France, both in terms of number of patients and hospital management.

These findings offer valuable insight on the current hospital management of VTE in cancer patients and guidance on future research on VTE prophylaxis in a very high-risk population, particularly in patients with active cancer. Together with close patient monitoring, it is essential to prevent these serious and sometimes lethal events.

## Supplementary Information

**Additional file 1:** **Supplement Table 1.** ICD-10 codes of the five cancers ofinterest. **Supplement Table 2.** ICD-10 codes of venous thromboembolismevents included in the study. **Supplement Table 3.** National healthcare system codes ofmedical procedures carried out to treat venous thromboembolism events includedin the study.

## Data Availability

The data supporting the study findings are part of the National health data system (SNDS, Système national des Données de Santé) and are available from the HDH (Health Data Hub https://www.health-data-hub.fr/). Restrictions apply to the availability of these data, which were used under the 006 framework (MR-006) developed by the French data protection authority (Commission Nationale de l’Informatique et des Libertés, CNIL).
