# Supplementary material for: Adult breast, lung, pancreatic, upper and lower gastrointestinal cancer patients with hospitalized venous thromboembolism in the national French hospital discharge database
Source: BMC Cancer. 2023 Jun 10;23:531. doi: 10.1186/s12885-023-10877-4 (PMC10257306; doi:10.1186/s12885-023-10877-4)
Supplement: Supplementary file 1 — Additional file 1: Supplement Table 1. ICD-10 codes of the five cancers ofinterest. Supplement Table 2. ICD-10 codes of venous thromboembolismevents included in the study. Supplement Table 3. National healthcare system codes ofmedical procedures carried out to treat venous thromboembolism events includedin the study. [file 12885_2023_10877_MOESM1_ESM.docx]

Supplement

**Adult breast, lung, pancreatic, upper and lower gastrointestinal cancer patients with hospitalized venous thromboembolism in the national French hospital** **discharge database**

F. Couturaud, I. Mahé, J. Schmidt, J-C. Gleize, T. Lafon, A. Saighi, F. Sedjelmaci, L. Bertoletti, P. Mismetti

[Supplement Table 1 ICD-10 codes of the five cancers of interest 2](#_Toc97801650)

[Supplement Table 2 ICD-10 codes of venous thromboembolism events included in the study 3](#_Toc97801651)

[Supplement Table 3 National healthcare system codes of medical procedures carried out to treat venous thromboembolism events included in the study 4](#_Toc97801652)

| Supplement Table 1 ICD-10 codes of the five cancers of interest |
| --- |

| ICD-10 code | Label |
| --- | --- |
| Upper gastrointestinal cancer | |
| C15 | Malignant neoplasm of oesophagus |
| C16 | Malignant neoplasm of stomach |
| D00 | Carcinoma in situ of oral cavity, oesophagus, and stomach |
| Lower gastrointestinal cancer | |
| C17 | Malignant neoplasm of small intestine |
| C21 | Malignant neoplasm of anus and anal canal |
| C26 | Malignant neoplasm of other and ill-defined digestive organs |
| D013 | Carcinoma in situ of other and unspecified digestive organs, Anus, and anal canal |
| D014 | Carcinoma in situ of other and unspecified digestive organs, Other and unspecified parts of intestine |
| D017 | Carcinoma in situ of other and unspecified digestive organs, Other specified digestive organs |
| D019 | Carcinoma in situ of other and unspecified digestive organs, Digestive organ, unspecified |
| C18 | Malignant neoplasm of colon |
| C19 | Malignant neoplasm of rectosigmoid junction |
| C20 | Malignant neoplasm of rectum |
| D010 | Carcinoma in situ of other and unspecified digestive organs, Colon |
| D011 | Carcinoma in situ of other and unspecified digestive organs, Rectosigmoid junction |
| D012 | Carcinoma in situ of other and unspecified digestive organs, Rectum |
| Pancreatic cancer | |
| C25 | Malignant neoplasm of pancreas |
| Lung cancer | |
| C33 | Malignant neoplasm of trachea |
| C34 | Malignant neoplasm of bronchus and lung |
| D021 | Carcinoma in situ of middle ear and respiratory system, Trachea |
| D022 | Carcinoma in situ of middle ear and respiratory system, Bronchus and lung |
| Breast cancer | |
| C50 | Malignant neoplasm of breast |
| D05 | Carcinoma in situ of breast |

# Supplement Table 2 ICD-10 codes of venous thromboembolism events included in the study

| ICD-10 code | Label |
| --- | --- |
| Venous thrombosis | |
| I63.6 | Cerebral infarction due to cerebral venous thrombosis, nonpyogenic |
| I67.6 | Nonpyogenic thrombosis of intracranial venous system |
| I80.0 | Phlebitis and thrombophlebitis of superficial vessels of lower extremities |
| I80.1 | Phlebitis and thrombophlebitis of femoral vein |
| I80.2 | Phlebitis and thrombophlebitis of other deep vessels of lower extremities |
| I80.3 | Phlebitis and thrombophlebitis of lower extremities, unspecified |
| I80.8 | Phlebitis and thrombophlebitis of other sites |
| I80.9 | Phlebitis and thrombophlebitis of unspecified site |
| I82.0 | Budd-Chiari syndrome |
| I82.1 | Thrombophlebitis migrans |
| O22.2 | Venous complications in pregnancy: Superficial Thrombophlebitis |
| O22.3 | Venous complications in pregnancy: Deep Thrombophlebitis |
| O22.5 | Venous complications in pregnancy: Cerebral venous Thrombosis |
| O87.0 | Venous complications in the puerperium: Superficial Thrombophlebitis |
| O87.1 | Venous complications in the puerperium: Deep Thrombophlebitis |
| O87.3 | Venous complications in the puerperium: Cerebral venous thrombosis |
| Embolism |  |
| I26.0 | Pulmonary embolism with mention of acute core pulmonale |
| I26.9 | Pulmonary embolism without mention of acute core pulmonale |
| I82.2 | Embolism and thrombosis of vena cava |
| I82.3 | Embolism and thrombosis of renal vein |
| I82.8 | Embolism and thrombosis of other specified veins |
| I82.9 | Embolism and thrombosis of unspecified vein |
| O88.2 | Obstetric embolism |

# Supplement Table 3 National healthcare system codes of medical procedures carried out to treat venous thromboembolism events included in the study

| CCAM code | Label |
| --- | --- |
| Thrombectomie | |
| DFFA001 | Embolectomie de l'artère pulmonaire, par thoracotomie avec CEC |
| DFFA002 | Embolectomie de l'artère pulmonaire, par thoracotomie sans CEC |
| DFNF002 | Thrombolyse mécanique ou thromboaspiration de l'artère pulmonaire, par voie veineuse transcutanée |
| Thromboaspiration | |
| DFNF002 | Thrombolyse mécanique ou thromboaspiration de l'artère pulmonaire, par voie veineuse transcutanée |
| DHNF001 | Thrombolyse mécanique ou thromboaspiration de la veine cave supérieure, par voie veineuse transcutanée |
| DHNF003 | Thrombolyse mécanique ou thromboaspiration de la veine cave inférieure, par voie veineuse transcutanée |
| EFJF001 | Thromboaspiration de la veine subclavière et/ou de la veine brachiocéphalique, par voie veineuse transcutanée |
| EGJF001 | Thromboaspiration de la veine rénale, par voie veineuse transcutanée |
| EGJF002 | Thromboaspiration de la veine iliaque externe et/ou de la veine iliaque commune, par voie veineuse transcutanée |
| EZJF001 | Thromboaspiration d'un accès vasculaire artérioveineux d'un membre avec dilatation intraluminale et pose d'endoprothèse, par voie vasculaire transcutanée |
| EZJF002 | Thromboaspiration d'un accès vasculaire artérioveineux d'un membre avec dilatation intraluminale sans pose d'endoprothèse, par voie vasculaire transcutanée |
| EPJB015 | Évacuation de thrombus de veine superficielle des membres ou du cou, par voie transcutanée |
| Fibrinolyse |  |
| DFNF001 | Fibrinolyse in situ de l'artère pulmonaire, par voie veineuse transcutanée |
| DHNF002 | Fibrinolyse in situ fémoro-ilio-cave, par voie veineuse transcutanée |
| DHNF004 | Fibrinolyse in situ de la veine cave inférieure, par voie veineuse transcutanée |
| DHNF005 | Fibrinolyse in situ de la veine cave supérieure, par voie veineuse transcutanée |
| DHNF006 | Fibrinolyse in situ fémoro-ilio-cave avec oblitération partielle de la veine cave inférieure [pose d'un filtre cave], par voie veineuse transcutanée |
| EFNF001 | Fibrinolyse in situ de la veine subclavière et/ou de la veine brachiocéphalique, par voie veineuse transcutanée |
| EGNF001 | Fibrinolyse in situ de la veine rénale, par voie veineuse transcutanée |
| EGNF002 | Fibrinolyse in situ de la veine iliaque externe et/ou de la veine iliaque commune, par voie veineuse transcutanée |
| EHNF001 | Fibrinolyse in situ de la veine porte et/ou de ses affluents ou d'un court-circuit [shunt] vasculaire portosystémique, par voie veineuse transcutanée |
| EJNF001 | Fibrinolyse in situ d'une veine du membre inférieur, par injection transcutanée dans une veine du dos du pied |
| EZNF001 | Fibrinolyse in situ d'un accès vasculaire artérioveineux d'un membre avec dilatation intraluminale sans pose d'endoprothèse, par voie vasculaire transcutanée |
| EZNF002 | Fibrinolyse in situ d'un accès vasculaire artérioveineux d'un membre avec dilatation intraluminale et pose d'endoprothèse, par voie vasculaire transcutanée |
| EZNF004 | Fibrinolyse in situ d'un accès vasculaire artérioveineux des membres, par injection intravasculaire transcutanée |
| Pose de filtre cave | |
| DHSF001 | Oblitération partielle temporaire de la veine cave inférieure, par voie veineuse transcutanée |
| DHSF002 | Oblitération partielle définitive de la veine cave inférieure, par voie veineuse transcutanée |
| DHNF006 | Fibrinolyse in situ fémoro-ilio-cave avec oblitération partielle de la veine cave inférieure [pose d'un filtre cave], par voie veineuse transcutanée. |

CCAM: Classification Commune des Actes Médicaux, medical procedures common classification
